# Abnormal myocardial work in children with Kawasaki disease

**DOI:** 10.1038/s41598-021-86933-5

**Published:** 2021-04-12

**Authors:** Jolanda Sabatino, Nunzia Borrelli, Alain Fraisse, Jethro Herberg, Elena Karagadova, Martina Avesani, Valentina Bucciarelli, Manjit Josen, Josefa Paredes, Enrico Piccinelli, Maraisa Spada, Sylvia Krupickova, Ciro Indolfi, Giovanni Di Salvo

**Affiliations:** 1 Department of Paediatric Cardiology, Royal Brompton Hospital, London, SW36NP UK; 2 National Heart and Lung Institute, Imperial College, London, UK; 3 Division of Cardiology, Department of Medical and Surgical Science, URT-CNR, Magna Graecia University, Catanzaro, Italy

**Keywords:** Cardiology, Cardiovascular diseases

## Abstract

Kawasaki disease (KD) can be associated with high morbidity and mortality due to coronary artery aneurysms formation and myocardial dysfunction. Aim of this study was to evaluate the diagnostic performance of non-invasive myocardial work in predicting subtle myocardial abnormalities in Kawasaki disease (KD) children with coronary dilatation (CADL). A total of 100 patients (age 8.7 ± 5 years) were included: 45 children with KD and CADL (KD/CADL) (Z-score > 2.5), 45 age-matched controls (CTRL) and, finally, an additional group of 10 children with KD in absence of coronary dilatation (KD group). Left ventricular (LV) systolic function and global longitudinal strain (GLS) were assessed. Global myocardial work index (MWI) was calculated as the area of the LV pressure-strain loops. From MWI, global Constructive Work (MCW), Wasted Work (MWW) and Work Efficiency (MWE) were estimated. Despite normal LV systolic function by routine echocardiography, KD/CADL patients had lower MWI (1433.2 ± 375.8 mmHg% vs 1752.2 ± 265.7 mmHg%, p < 0.001), MCW (1885.5 ± 384.2 mmHg% vs 2175.9 ± 292.4 mmHg%, p = 0.001) and MWE (994.0 ± 4.8% vs 95.9 ± 2.0%, p = 0.030) compared to CTRL. Furthermore, MWI was significantly reduced in children belonging to the KD group in comparison with controls (KD: 1498.3 ± 361.7 mmHg%; KD vs CTRL p = 0.028) and was comparable between KD/CADL and KD groups (KD/CADL vs KD p = 0.896). Moreover, KD/CADL patients with normal GLS (n = 38) preserved significant differences in MWI and MCW in comparison with CTRL**.** MWI, MCW and MWE were significantly reduced in KD children despite normal LVEF and normal GLS. These abnormalities seems independent from CADL. Thus, in KD with normal LVEF and normal GLS, estimation of MWI may be a more sensitive indicator of myocardial dysfunction.

## Introduction

Kawasaki disease (KD) represents the leading cause of acquired heart disease in children in developed countries^1^. It is an acute inflammatory disorder of unknown etiology, which predominantly affects the coronary circulation leading to aneurysm formation^1–4^.

In addition, late myocardial abnormalities and biopsy proven myocardial fibrosis have been found up to 11 years after the acute disease, even in presence of normal left ventricular (LV) systolic function^5,6^ and normal coronary arteries^7^.

Although KD carries with it some myocardium inflammation, routine echocardiographic measurements of LV systolic function are often normal, limiting their usefulness^8^.

Speckle tracking echocardiography (STE) has previously identified patients with coronary disease with good sensitivity^9^. Moreover, recent studies have demonstrated abnormal strain in patients with KD, despite normal left ventricular ejection fraction^10,11^.

Unfortunately, even if lower than standard echocardiographic parameters of LV function, STE indices are load dependent^12,13^, and may be inaccurate in specific settings^14^.

An alternative proposed approach is the estimation of myocardial work, which is defined as cardiac output multiplied by aortic pressure, and it can be calculated as the area within the pressure–volume loop during one cardiac cycle^15^. Nevertheless, myocardial work estimation has been evaluated invasively in the past decades, being consequently not feasible in routine assessments.

Recently, a non-invasive method has been introduced to quantify global and segmental myocardial work, that match longitudinal strain with standardized LV pressure-curves, adjusted to brachial cuff pressure and valvular events^16,17^.

Non-invasive MW showed a strong correlation with invasive work measurements in recent validation studies. Moreover, Boe et al.^18^ demonstrated the ability of regional non-invasive myocardial work index (MWI) to identify acute coronary occlusion in patients with NSTE-ACS.

We hypothesized that, in children with KD and coronary dilatation, despite normal LV systolic function measured by two-dimensional ejection fraction (EF), indices of myocardial work will be impaired.

Therefore, the aim of the present study was to investigate, for the first time, the ability of non-invasive myocardial work indices to evaluate subtle changes in myocardial function among KD patients with coronary dilatation and normal LVEF.

## Results

### Study population

Clinical and standard echocardiographic characteristics of the study sample are presented in Table 1. A total of 100 patients (64 males, age 8.7 ± 5 years) were included in our study. Among the children admitted to our Institution with a diagnosis of KD during the study time frame, 45 patients ^31males^ with coronary artery dilatation (Z-score > 2.5) were selected and included in the KD/CADL group. Among those, 38 had coronary aneurysms and 13 presented giant coronary aneurysms. The average maximal coronary diameter in the KD/CADL group was 6.5 ± 3.8 mm, while the average Z-score was 8.9 ± 6.9. Twenty-five children presented dilatation of LMCA (left main coronary artery); 10 of LAD; 25 of RCA. Also, 3 children presented dilatation of LMCA, LAD and RCA; 14 of LMCA and RCA; 2 of LAD and RCA. No patients presented thrombus or stenosis of the coronary artery. KD/CADL patients were compared with 45 (31 males) age- and weight-matched controls (CTRL) and to an additional group of 10 children (2 males) with Kawasaki disease in absence of coronary dilatation (KD group) (Table 1).

**Table 1:** Baseline characteristics of clinical and echocardiographic variables.

Clinical and echocardiographic variables	Baseline characteristics			
	KD/CADL (N = 45)	KD (N = 10)	CTRL (N = 45)	P = (KD/CADL vs CTRL)
Age (years)	8.0 ± 5	6.8 ± 5	9.7 ± 5	0.695
Age range (years)	0–16	1–16	0–16	N/A
Male, n (%)	31 (69)	2 (20)	31 (69)	N/A
Body surface area (BSA) (m^2^)	1.02 ± 0.41	0.93 ± 0.52	1.17 ± 0.47	0.599
SBP (mmHg)	106 ± 15^§^	105 ± 13	117 ± 11	0.001
DBP (mmHg)	64 ± 9^§^	65 ± 4	70 ± 11	0.002
HR (bpm)	100 ± 25^§^	94 ± 19	81 ± 22	0.002
LVEDD (mm)	36.0 ± 8.2	32.5 ± 9.1	35.5 ± 8.3	0.899
LVEDD Z score	− 0.36 ± 1.96	− 0.45 ± 1.24	− 0.85 ± 1.90	0.356
LVESD (mm)	24.0 ± 8.2	22.0 ± 6.4	24.0 ± 6.4	0.919
LVESD Z score	+ 0.11 ± 1.56	0.13 ± 1.28	− 0.58 ± 1.80	0.135
LVEF (%)	63.4 ± 4	63.0 ± 6	63.5 ± 5	0.738
LMCA aneurysm, n (%)	25 (3.3)	N/A	N/A	N/A
LAD aneurysm, n (%)	10 (0.5)	N/A	N/A	N/A
RCA aneurysm, n (%)	25 (2.8)	N/A	N/A	N/A

Values are mean (SD), or n (%).

*KD/CADL* group of children with Kawasaki disease and coronary aneurysms, *KD* group of children with Kawasaki disease without coronary aneurysms, *LVEDD* left ventricular end diastolic diameter, *LVESD* left ventricular end systolic diameter, *LVEF* left ventricular ejection fraction, *BSA* body surface area, *HR* heart rate, *SBP* systolic blood pressure, *DBP* diastolic blood pressure, *LMCA* left main coronary artery, *LAD* left anterior descending artery, *RCA* right coronary artery.

^§^p < 0.05 compared to CTRL.

No significant differences were observed between KD/CADL group and CTRL with respect to age, sex, and body surface area. Systemic blood pressure was observed to be lower in KD/CADL group compared to CTRL, as regard both SBP and DBP (SBP in KD/CADL: 106 ± 15 mmHg vs CTRL: 117 ± 11 mmHg, p = 0.001; DBP in KD/CADL: 64 ± 9 mmHg vs CTRL: 70 ± 11 mmHg, p = 0.002). HR was found to be significantly higher in KD/CADL group compared to CTRL (100 ± 25 bpm vs 81 ± 22 bpm, p = 0.002). Patients were not on any medication, apart from aspirin or warfarin regimens. The average time between KD onset and the assessment of myocardial work by echocardiography in KD children was 2 ± 2 years (time range: 16–2855 days).

### Standard echo parameters (Table 1)

LV dimensions were comparable in KD/CADL group and CTRL as regard to the absolute values (LVEDD in KD/CADL: 36.0 ± 8.2 vs CTRL: 35.5 ± 8.3, p = 0.899; LVESD in KD/CADL: 24.0 ± 8.2 vs CTRL: 24.0 ± 6.4, p = 0.919) or normalized for the Z score (LVEDD Z score in KD/CADL: -0.36 ± 1.96 vs CTRL: − 0.85 ± 1.90, p = 0.356; LVESD Z score in KD/CADL: + 0.11 ± 1.56 vs CTRL: − 0.58 ± 1.80, p = 0.135). LV dimensions of children with KD in absence of coronary aneurysms are presented in Table 1.

**Figure 1:**
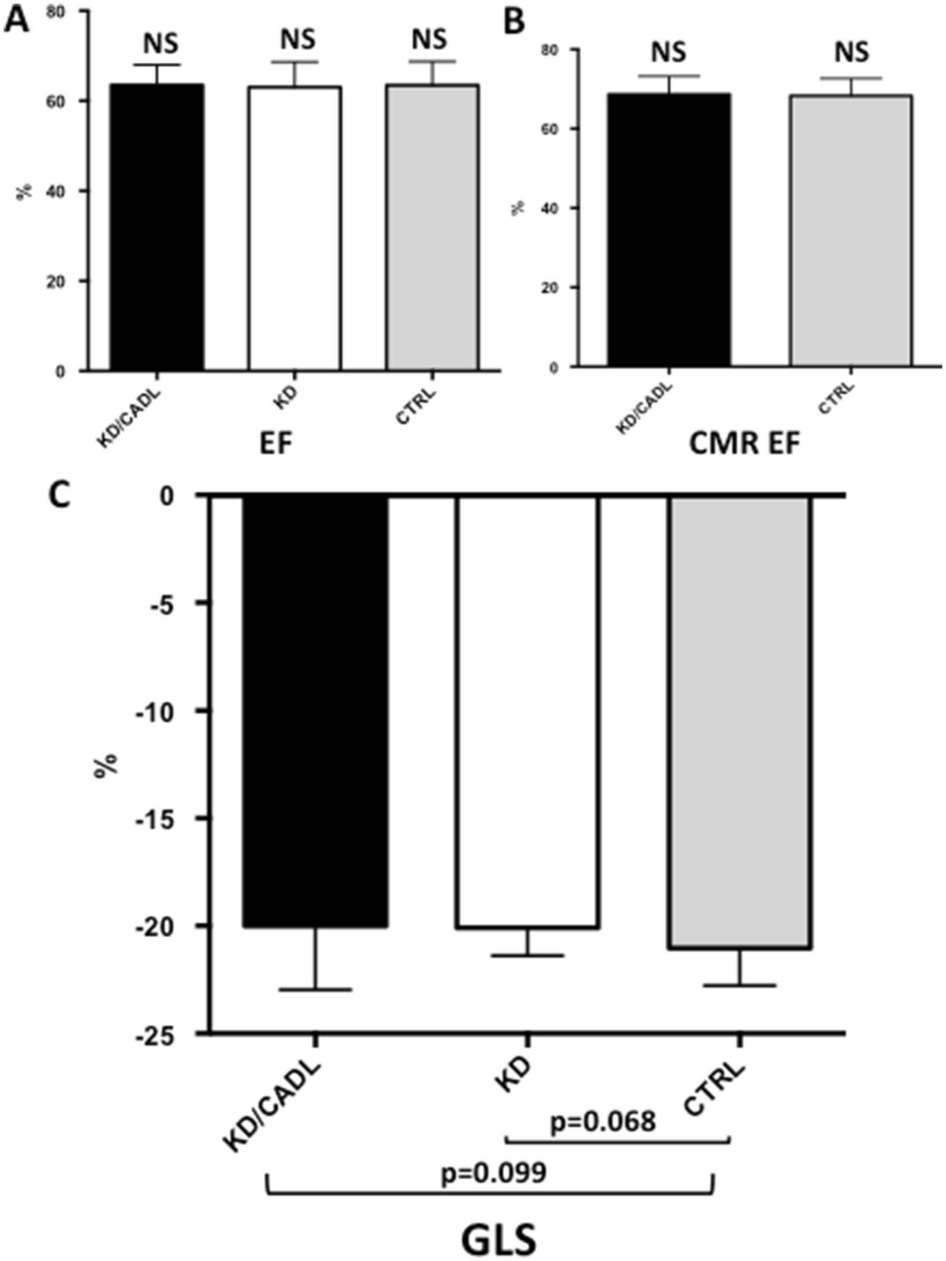
The figure shows that LV ejection fraction measured either by echocardiogram **(A)** or CMR **(B)** did not differ significantly in children with Kawasaki disease compared to CTRLs. Global longitudinal strain values were within the normal range both in the KD/CADL and in the KD group and comparable to CTRLs **(C)**. Graphs in this figure were drawn by using Past software (version 4.02). *CMR* cardiovascular magnetic resonance, *KD* Kawasaki disease, *CTRLs* controls.

No significant differences were observed in LVEF values between KD/CADL children and CTRL (KD: 63.4 ± 4% vs CTRL: 63.5 ± 5%, p = 0.738) (Fig. 1A).

### CMR assessment

CMR was performed in 13 CTRL and in 13 patients from KD group for a clinical indication: ten for the presence of giant coronary aneurysms, the remaining three patients for the presence of symptoms (angina or tiredness) on exercise. Coronary arteries were confirmed to be enlarged by the CMR findings. No patients presented thrombus or stenosis of the coronary artery.

Among the patients who underwent CMR, nine received adenosine stress for a clinical indication: six patients presented with giant coronary aneurysms and three were experiencing symptoms on exercise. LV ejection fraction measured by CMR assessment did not differ in KD/CADL children compared to CTRLs (KD/CADL: 68.6 ± 4% vs CTRL: 65.8 ± 5%, p = 0.082) (Fig. 1B).

Moreover, 2 out of 13 patients from the KD/CADL group, who received gadolinium injection, presented with LV LGE (4 LV segments total). Only one child out of nine from the KD/CADL group, revealed perfusion defects after adenosine stress.

### Speckle tracking echocardiography

Global longitudinal strain values were within the normal range both in the KD/CADL and in the KD group, and we found no significant differences compared to CTRL (KD/CADL: − 20.0 ± 2.9%; KD: − 20.1 ± 1.3%; CTRL: − 21.0 ± 1.7%; KD/CADL vs CTRL p = 0.099, KD vs CTRL p = 0.068) (Fig. 1C). Thirty-eight out of 45 children in KD/CADL group presented GLS ≥ − 19%, and all the KD without CADL.

### Myocardial work analysis

**Figure 2:**
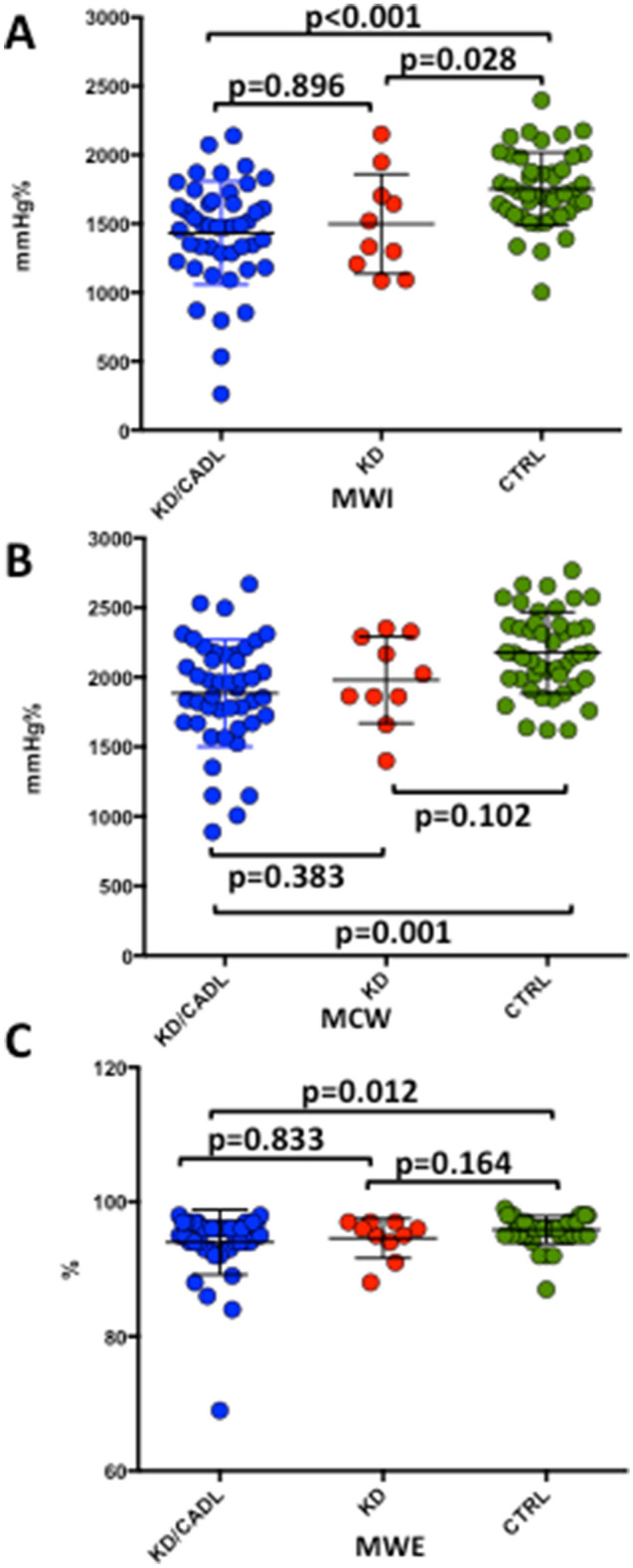
The graphs show global MWI **(A)**, MCW **(B)** and MWE **(C)** significantly reduced in children belonging to the KD/CADL group compared to controls. Likewise, KD group shows lower MWI values compared with CTRL. Graphs in this figure were drawn by using Past software (version 4.02). *MWI* myocardial work index, *MCW* myocardial constructive work, *MWE* myocardial wasted work.

**Figure 3:**
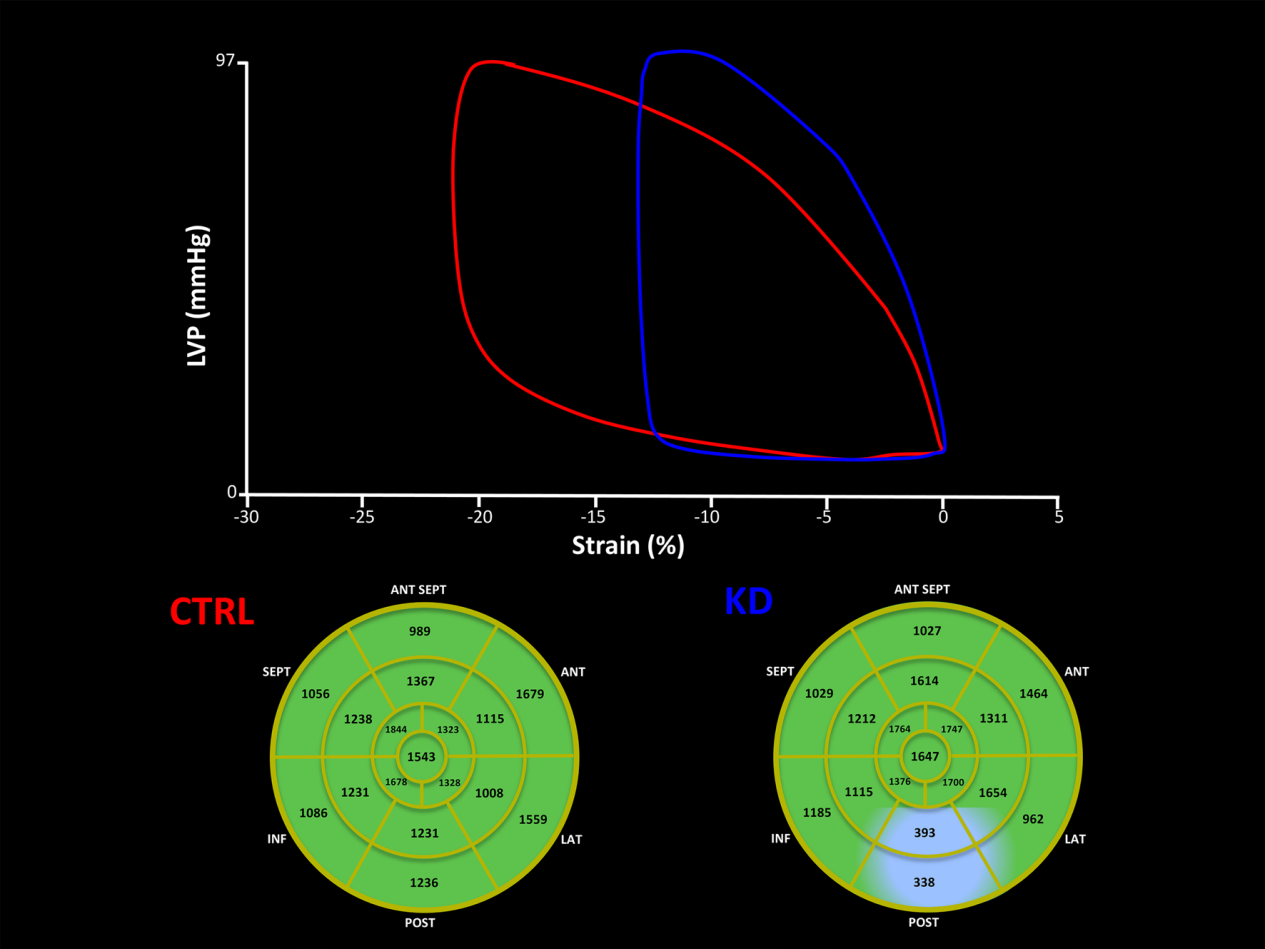
The figure shows an example of the pressure strain loops and the integrated area.

Global MWI (KD/CADL: 1433.2 ± 375.8 mmHg%; KD: 1498.3 ± 361.7 mmHg%; CTRL: 1752.2 ± 265.7 mmHg%; KD/CADL vs CTRL p < 0.001; KD vs CTRL p = 0.028) was significantly reduced in children belonging both to the KD/CADL or the KD group compared to controls (Figs. 2A, 3). Also, global MWE (94.0 ± 4.8% vs 95.9 ± 2.0%, p = 0.012), MCW (1885.5 ± 384.2 mmHg% vs 2175.9 ± 292.4 mmHg%, p = 0.001) were significantly reduced in KD/CADL group compared to controls (Fig. 2B,C). On the contrary, MWW was not significantly increased in KD/CADL compared to CTRL (p = 0.080).

**Figure 4:**
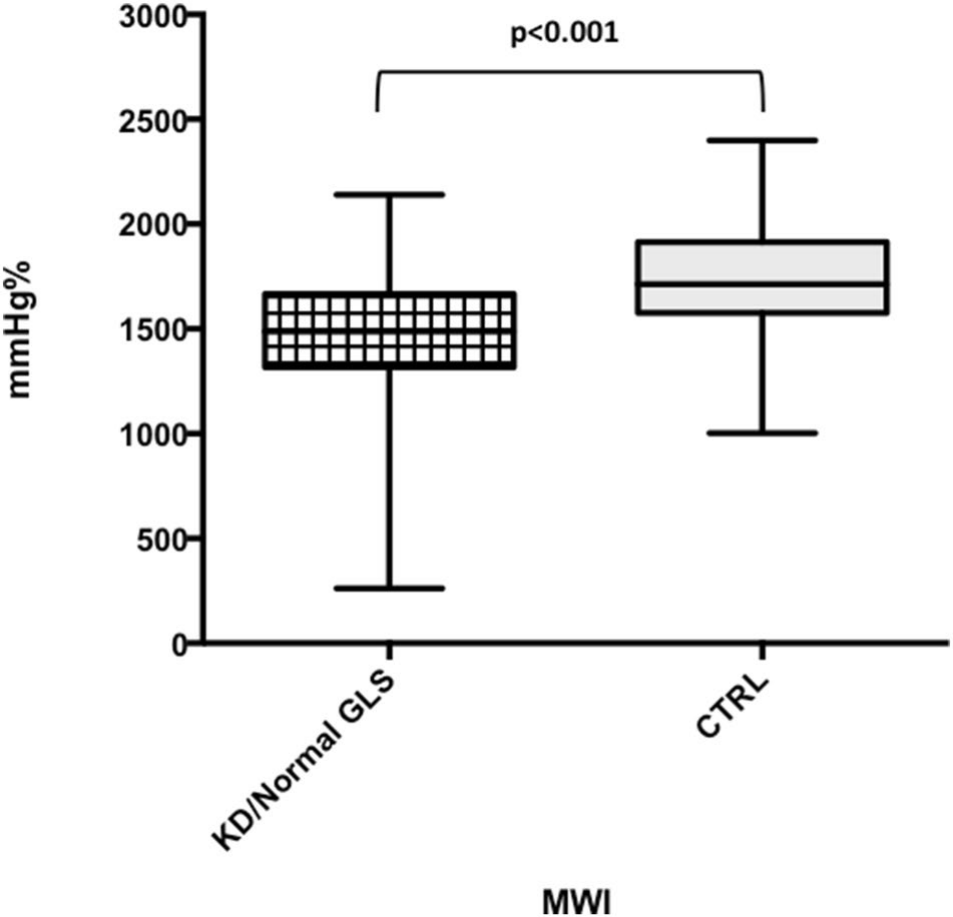
KD/CADL patients with normal GLS presented significant differences in MWI values compared to controls. Graphs in this figure were drawn by using Past software (version 4.02).

When KD/CADL patients with normal GLS (≥ − 19%) were analysed separately (n = 38), they maintained a significant difference in MWI and MCW in comparison with controls (MWI: 1477 ± 345 mmHg% in KD/CADL vs 1751 ± 263 mmHg% in CTRL, p < 0.001; MCW: 1950 ± 325 mmHg% in KD/CADL vs 2170 ± 291 mmHg% in CTRL, p = 0.002) (Fig. 4).

In KD/CADL group, children with GLS reduction presented increased coronary size compared to children without GLS reduction (average maximal coronary diameter: 10.4 ± 5.7 mm vs 5.8 ± 2.9, p = 0.002; average Zscore 15.2 ± 10.1 vs 7.7 ± 5.6, p = 0.007). CMR data are available only in two children with GLS reduction: one child presented CMR positive for LGE and adenosine inducible ischemia, while the other child presented normal CMR findings. Also, MW indices were reduced in children with GLS < − 19% compared to children with normal strain (Table 2).

**Table 2:** Echocardiographic variables of KD/CADL children with normal strain or strain reduction.

	Maximal coronary diameter (mm)	Maximal coronary diameter (Z score)	MWI	MCW	MWW	MWE
Normal GLS (n = 38)	5.8 ± 2.9	7.7 ± 5.6	1478 ± 345	1951 ± 325	90 ± 56	95 ± 3
Reduced GLS (n = 37)	10.4 ± 5.7	15.2 ± 10.1	1191 ± 468	1532 ± 508	112 ± 50	89 ± 9

Values are mean (SD).

*GLS* global longitudinal strain, *MWI* myocardial work index, *MCW* myocardial constructive work, *MWW *myocardial wasted work, *MWE* myocardial work efficiency.

### MWI correlations

Modest correlations were found between the LMCA size and MWI (r = − 0.412; p < 0.001), MCW (r = − 0.437; p < 0.001), MWE (r = − 0.370; p = 0.001) or GLS (r = 0.392; p = 0.001). Also, we observed modest correlations between the LAD size and the same parameters (MWI: r = − 0.398; p = 0.002; MCW: r = − 0.367; p = 0.004; MWE: r = − 0.488; p < 0.001; GLS: r = 0.391; p = 0.002), but not significant correlations between these and the RCA size.

Weak correlations were identified between MWI (r = − 0.240; p = 0.022), MCW (r = − 0.263; p = 0.012), MWE (r = − 0.275; p = 0.008) or GLS (r = 0.267; p = 0.011) and the aneurysm dimensions assessed by z-score. Also, MWI (r = − 0.281; p = 0.007), MCW (r = − 0.264; p = 0.011), MWE (r = − 0.219; p = 0.037) but not GLS (r = 0.170; p = 0.108), were very weakly correlated with the number of coronaries involved in the disease.

Furthermore, classical LV ejection fraction assessed either by echocardiogram or CMR did not significantly correlate with the LMCA (r = 0.132; p = 0.522 and r = − 0.256; p = 0.540), LAD (r = − 0.022; p = 0.944 and N/A) or RCA sizes (r = − 0.346; p = 0.060 and r = − 0.402; p = 0.174) or with the number of coronaries involved in the disease (r = 0.055; p = 0.721 and r = − 0.256; p = 0.540).

### Reproducibility analysis

Intra-observer variability was very good for longitudinal strain (7 ± 7%; ICC: 0.92) and good for MWI (13 ± 4%; ICC: 0.89). Inter-observer variability was also very good for longitudinal strain (7 ± 8%; ICC: 0.93) and good for MWI (15 ± 6%; ICC: 0.87).

## Discussion

This is the first study assessing the diagnostic performance of non-invasive myocardial work in a cohort of children and young patients with KD with and without coronary dilatation. Our data, firstly, demonstrated that non-invasive myocardial work, and its derived indices, is reduced in patients with KD even in absence of coronary aneurysm. MWI could detect subtle myocardial abnormalities better than GLS and LVEF.

### Myocardial function in children with KD

Myocardial involvement in KD has been extensively elucidated with several studies showing the characteristic abnormal histology, which appears to be similar in all the patients with KD^5–7^. Kang et al. demonstrated impaired left atrial reservoir function in children with KD in the acute phase^19^.

McCandless et al.^10^ using strain and strain rate showed that children with KD (n = 32), despite normal LV systolic function values assessed by routine echocardiographic measurements, had reduced longitudinal myocardial deformation properties.

Moreover, Sanchez et al.^11^ have recently demonstrated that children in the convalescent/chronic phase of KD (n = 67) are characterized by a subtle reduction in strain rate compared to the acute phase, that it is more pronounced in patients with coronary aneurysm than in those without.

In agreement with these studies^10,11^, we have confirmed a reduction of global longitudinal strain even if not statistical significant, in a sample of 45 children with established diagnosis of KD complicated by coronary dilatation when compared to CTRLs and also in group of ten children with KD in absence of coronary aneurysms.

Our study was the first to assess non-invasive MWI and its derived indices in children with KD complicated by CADL. We detected an abnormally reduced MWI, MCW and MWE in the group of patients with KD and CADL, compared to the CTRL.

On the other hand, LV ventricular ejection fraction, measured either by echo or by CMR, showed no significant differences between KD group and CTRL in our cohort of patients.

Only two (15.4%) out of 13 patients with KD, who underwent CMR with gadolinium injection, presented LV LGE (4 LV segments total). Similarly only one (11.1%) child out of nine who underwent CMR adenosine, revealed perfusion defects.

In our study cohort, we observed a significant impairment of MWI also in a group of 10 KD children without CADL. Furthermore, we found modest correlations among the aneurysm size on LMCA, LAD and MW indices and not significant correlations between these and the RCA size. Also, we identified weak correlations between MWI (r = − 0.240; p = 0.022), or its derived indices (MCW: r = − 0.263; p = 0.012, MWE: r = − 0.275; p = 0.008), and the aneurysms dimensions assessed by z-score. Finally, MWI (r = − 0.281; p = 0.007), MCW (r = − 0.264; p = 0.011), MWE (r = -0.219; p = 0.037), presented a weak correlation with the number of coronaries involved in the disease.

Indeed, a previous study of Ai-Min Liu et al.^20^, performed on endo-myocardial biopsy specimens obtained during follow-up from 54 patients who had typical clinical manifestations of KD, showed significantly increased incidences of myocardial hypertrophy, disarray of myocardial fibres, and micro-vascular lesions in the group of patients with KD and coronary artery lesions, compared to those without.

In our study, in a subgroup of children with KD complicated by CADL but with normal GLS (n = 38), MWI was found to be still reduced compared to CTRL, being able to distinguish the former from the healthy ones.

Hence, measuring global non-invasive MWI seems more appropriate to study cardiac function in KD children with normal LVEF and GLS.

### Myocardial work and ventricular function

Myocardial work, calculated from LV pressure/volume or pressure/length loops during cardiac catheterization procedures, has been validated as a reliable marker of ventricular contractility for 40 years^21–23^. Also, it has been recently demonstrated to show similar physiological information as pressure/strain loops^24^.

In this setting, a study from Russell et al.^16^ introduced a method to calculate the non-invasive MW, using speckle tracking analysis and estimating LV pressure from brachial artery cuff pressure.

Moreover, the NORRE sub-study provided reference ranges in adults for non-invasive MW in a multicentre study design^25,26^. In this setting, Manganaro et al., demonstrated a good reproducibility for the assessment of MW, further supporting its potential use in clinical practice.

To date, MW has been evaluated as a reliable predictor of response to CRT in patients with heart failure^17,27^. Most recently, Chan et al.^28^ reported data of MW indices in patients with hypertension, ischaemic and not-ischaemic dilated cardiomyopathy.

Furthermore, a previous study from our group demonstrated that the measurement of MW indices is a sensitive and early marker of myocardial ischemia during transient acute coronary occlusion^29^.

### Effects of blood pressure and heart rate on myocardial work indices

As described above, myocardial work indices are based on strain and blood pressure. Strain is afterload dependent. For this reason, any increase in afterload may cause a strain reduction in presence of normal contractility^12^. In our study, KD patients showed reduced MWI with significantly reduced BP. In states of normal contractility, in presence of reduced BP one would expect strain to be higher and so MWI. The fact that, in our study, MWI is reduced in KD patients, despite a reduced BP, strongly suggests that the physiologic difference is due to a reduced myocardial deformation. For this reason, we believe, that the lower BP we found in KD patients is not a confounder, but it rather strengths the physiological value of our findings.

Regarding HR, it was found to be significantly higher in KD group compared to CTRLs (100 ± 25 bpm vs 81 ± 22 bpm, p = 0.002).

However, according to the Treppe phenomenon, the increase in HR in normal inotropic states, leads to an increase in cardiac contractility until a certain threshold, and then the inotropic effect decreases. Accordingly, Davidavicius et al.^30^ demonstrated that peak systolic longitudinal strain showed a biphasic response to HR, initially increasing in response to early physiological heart rate increase (e.g. in the setting of exercise), and then remaining constant or falling at HR > 150 bpm.

In our study in KD patients with significantly lower BP and an average HR of 100 ± 25 bpm, it could be expected that longitudinal strain and, then, myocardial work would be higher, in presence of normal contractility. The fact that MWI is reduced despite an increase in HR, strongly suggests that the physiologic difference between KD and CTRL might be due to a reduced myocardial contraction.

### Clinical application

All the published studies have already demonstrated compelling data, including decreased LV longitudinal systolic strain and reduced mitral and septal annular early diastolic tissue velocities in patients with KD.

Even though the actual aetiology of altered LV mechanics in these patients remains unclear, it is tempting to speculate that structural alterations of the myocardium might play a contributory role. For this reason, the AHA algorithm, designed to improve early diagnosis for incomplete cases of KD, uses echocardiographic evidence of decreased systolic function as supporting criteria. Our study demonstrated for the first time that MW indices are reduced in KD patients with CADL even in presence of normal LVEF and LV GLS. Reduced non-invasive MW of the LV, thus, may be a more robust indicator of myocardial involvement in KD according to our data in the convalescent/chronic phase of KD complicated by CADL in children.

### Myocardial work in paediatric age

This is the first clinical application of MW in paediatric age. We provided, for the first time, paediatric normal values for MW indices in 45 healthy children. In our experience the feasibility of this technique is excellent, because 2-dimensional imaging quality in paediatrics is generally higher than adults. Indeed, in this study no subject was excluded because of poor image quality. On the other side, intra and inter-observer variability for MWI was relatively higher than previously rates published in adults^18^. This can be partially explained by the fact that we did not perform any specific acquisition for valvular timings and this may be particularly relevant in paediatric patients in presence of relatively high heart rate.

### Study limitations

This study carries several limitations.

First of all, it does not provide prospective long-term follow-up data, in order to establish whether patients with worse non-invasive MW might have worse clinical outcome. Thus, further larger prospective multicentre studies would be needed to answer this question.

Furthermore, we could not correlate MW indices with CMR findings of LV LGE or perfusion defects after adenosine stress, since only three patients in the KD group presented those alterations.

Moreover, the average time between KD onset and the assessment of myocardial work by echocardiography in KD children was 2 ± 2 years (time range: 16–2855 days). However, ultrastructural studies on endomyocardial biopsy specimens demonstrated that the coronary microvascular lesions in patients with KD, characterized by microvascular dilatation or endothelial cell injury, persisted after convalescent stage even up to 23 years^20^.

Finally, genetic testing results were not included in the present study.

## Conclusions

The estimation of myocardial work by pressure-strain loops is a novel tool for the evaluation of patients with KD. MWI, MCW and MWE were significantly reduced in KD patients with dilated coronaries. In KD patients with normal GLS, estimation of MWI, MCW and MWE may be a sensitive indicator of myocardial dysfunction in KD.

## Methods

All methods were performed in accordance with the relevant guidelines and regulations.

### Study population

The study population comprised 100 children (mean age 8.7 ± 5 years, Table 1), identified from the institutional database of the Royal Brompton Hospital’s Paediatric Kawasaki Service, from January 2017 to June 2019. The study protocol has been approved by local Research Committee (Royal Brompton Hospital Institutional Review Committee). A written informed consent has been obtained from the parents.

We retrospectively included paediatric patients with Kawasaki disease in convalescent/chronic phase complicated by coronary dilatation (z-score > 2.5) of at least one vessel. Patients during the acute phase of KD were excluded from this study. The acute phase was defined as follow: from day 1 of illness through day 14 of illness; day 1 of illness was the first day of fever as documented in the admission note. Patients with atypical/incomplete KD and those with delayed treatment (> 10 fever days) were excluded. Only KD patient with a normal ejection fraction were included in the study (LVEF ≥ 55%). The control population consisted of age- and gender-matched children who underwent echocardiography for murmur evaluation and they were found to have no cardiac abnormalities. Finally, we included an additional group of 10 children with Kawasaki disease in absence of coronary dilatation (KD group) (Table 1).

### Echocardiography and definitions

All the patients underwent transthoracic echocardiography, according to the Royal Brompton Hospital standard protocol^31^, using a GE E95 ultrasound system (GE Healthcare, Horten, Norway). Echocardiograms with inadequate images of coronary arteries or the LV myocardium were excluded from the analysis.

For the left anterior descending, left main and right main coronary arteries, serial echocardiogram measurements of vessel diameters were converted into BSA-adjusted Z-scores using published normal regression equations. Coronary artery dilatation (CADL) was defined as coronary artery diameter z-score > 2.5. Aneurysm was defined as a coronary diameter between 4 and 8 mm. For children under the age of 5, a diameter of > 3 mm was considered an aneurysm. Giant aneurysms were defined by a coronary diameter above 8 mm, in all age groups^2^. Chamber size and function were assessed as previously described^31^ and according to the latest guidelines^32^. Values for LV diameters and thickness were adjusted for body size and age expressed as z-scores according to the Boston Children’s Hospital z-score system^33^.

### Speckle tracking echocardiography

Global longitudinal strain was assessed as previously described^30^. Additional information on speckle tracking analysis and LV Ejection Fraction (EF) methods is available in our Supplementary Data.

### Myocardial work

Myocardial work index (MWI), and its derived indices, were estimated using a customized software (GE-Healthcare)^15–18^, represented by the latest vendor-specific version of the Automated Function Imaging software (EchoPAC Version 202), and allowing the evaluation of myocardial work as a function of time during the whole cardiac cycle. The authors obtained simultaneously blood pressure by a cuff manometer at the time of 4ch/3ch/2ch image two-dimensional echocardiographic views acquisition^15–18^.

The MW module (GE-Healthcare) in AFI asks the user to provide blood pressure and valvular event times as input to the myocardial work estimation. A bull’s eye (Fig. 3) with the segmental MWI values and global values is then provided. A 17-segment LV model was used for this purpose.

Derived indices from MWI are defined as follows:

- Myocardial Constructive work (MCW): work performed by a segment during shortening in systole adding negative work during lengthening in IVR;
- Myocardial Wasted work (MWW): negative work performed by a segment during lengthening in systole adding work performed during shortening in IVR;
- Myocardial work efficiency (MWE): constructive work divided by the sum of constructive and wasted work (0–100%).

In our experience the added time to perform MW study by expert sonographers is 3 ± 2 min.

Further information on “Myocardial work” methods is provided in our Supplementary Data.

### Cardiovascular magnetic resonance analysis

Cardiovascular Magnetic Resonance (CMR) images were evaluated for the presence of aneurysms of the main epicardial coronary arteries. The right coronary artery, left main coronary artery, left anterior descending coronary artery, and left circumflex coronary artery were assessed. These were also evaluated for the presence of thrombi. Thrombi were diagnosed as a low-signal mass against the wall of the aneurysmal coronary artery, with a filling defect in the aneurysm in both the coronary sequences and the sequences with delayed enhancement.

Myocardial wall motion was qualitatively analysed^18^ and classified as normal or abnormal, which includes hypokinetic, dyskinetic, or akinetic wall motions. A 17-segment model was used for analysis. Segmental myocardial first-pass perfusion after adenosine stress was evaluated qualitatively and assessed as normal or as revealing a perfusion defect. Delayed contrast-enhanced images were evaluated visually for areas of late gadolinium enhancement (LGE) indicating scar due to myocardial infarction. Delayed hyperenhancement were categorized as subendocardial (≤ 50% wall thickness) or transmural (> 50% of wall thickness).

### Statistical analysis

Statistical analysis was performed^34^ using a standard statistical software program (SPSS ver. 20.0, IBM, Chicago, IL) and is extensively described in Supplementary Data.

### Ethics approval

The study protocol has been approved by local Research Committee.

### Consent to participate

Written informed consent was obtained from the parents.

## Supplementary Information

Supplementary Information.
