# Supplementary material for: Abnormal myocardial work in children with Kawasaki disease
Source: Sci Rep. 2021 Apr 12;11:7974. doi: 10.1038/s41598-021-86933-5 (PMC8042008; doi:10.1038/s41598-021-86933-5)
Supplement: Supplementary file 1 — Supplementary Information. [file 41598_2021_86933_MOESM1_ESM.docx]

**Abnormal Myocardial Work in Children with Kawasaki Disease.**

Authors: Jolanda Sabatino, MD, PhD^1,2^, Nunzia Borrelli, MD^1,2^, Alain Fraisse, MD^1,2^, Jethro Herberg MD^1,2^, Elena Karagadova^1,2^, Martina Avesani, MD^1,2^, Valentina Bucciarelli, MD^1,2^, Manjit Josen^1,2^, Josefa Paredes^1,2^, Enrico Piccinelli, MD^1,2^, Maraisa Spada, MD^1,2^, Sylvia Krupickova, MD^1,2^, Ciro Indolfi, MD^3^ and Giovanni Di Salvo, MD, PhD^1,2^.

**Affiliations:**

1. Department of Paediatric Cardiology, Royal Brompton Hospital, London, UK

2. National Heart and Lung Institute, Imperial College, London, UK

3. Division of Cardiology, Department of Medical and Surgical Science, URT-CNR, Magna Graecia University, Catanzaro, Italy.

**Supplementary Data**

**Supplementary Methods**

*Speckle tracking echocardiography*

Two-dimensional (2D) 4-chambers, 3-chambers and 2-chambers apical views were acquired with a frame rate ≥60 frames/sec and, then, transferred to a dedicated workstation for the offline analysis (EchoPAC, GE Healthcare). The recordings were processed using an acoustic-tracking software (EchoPAC version 112.99, Research Release, GE Healthcare), which allowed an offline semi-automated analysis of speckle-based strain. To calculate the LV global longitudinal strain (GLS), a line was traced along the LV endocardium's inner border in each of the three apical views, and a region of interest, between the endocardial and epicardial borders, was recognized by the EchoPAC software. The region of interest was, then, adjusted to ensure that the wall thickness was incorporated in the analysis, avoiding the pericardium and following myocardial motion. Results of segmental and global LV longitudinal peak systolic strain values were then provided by the software. For all the enrolled patients the image quality was excellent, and no LV segments were precluded from analysis.

From the same views we also evaluated LV Ejection Fraction (EF) by using Simpson’s method. The four-chambers and two-chambers views in end-diastole and end-systole were used. Once the endocardial border was traced, the echo machine software automatically calculated the EF value.

*Myocardial work*

As previously described by Russell et al (16), a combination of non-invasively estimated LV pressure curve with LV strain measurements should be used to assess myocardial work.

Peak systolic LV pressure was assumed to be equal to peak arterial pressure measured by a cuff manometer and assumed to be uniform throughout the ventricle. The non-invasive LV pressure curve was then acquired using an empiric and normalized reference curve that was adapted to the length of the isovolumetric and ejection phases, defined by the timing of aortic and mitral valve events by echocardiography (15-18).

Strain and pressure data were coordinated using the onset of R wave in the electrocardiogram. Myocardial work was then quantified by calculating the rate of segmental shortening by differentiation of the strain curve and multiplying this value with instantaneous LV pressure. This product is a measure of instantaneous power, which was integrated over time to obtain myocardial work as a function of time in systole, which is defined as the time interval from mitral valve closure to mitral valve opening.

Indeed, global (GlW), positive (GlWp) and negative (GlWn) work parameters are generated as mean values over all segments, according to the following formula (15):

GlW_p_ = 1/N ∑^N^_s=1_ W_p,s_, GlW_n_ = 1/ N∑^N^_s=1_ W_n,s_, GlW = GlW_n_/GlW_p_, where N is the total number of segments.

At the time of LV ejection period, work achieved by the myocardium during segmental elongation produces energy loss, which represents wasted work (MWW) of that segment. Myocardial work achieved during segmental shortening represented constructive work (MCW) of that segment. At the time of isovolumetric relaxation there is an inversion of those definitions so that myocardial work during shortening represents segmental WW and work during lengthening represents segmental MCW. By averaging segmental MCW and MWW, global MCW and MWW were estimated for the entire LV (15-18).

*Statistical analysis*

Continuous variables were expressed by mean and standard deviation. Categorial variables were expressed as numbers and percentages. Normality was assessed using Q-Q plots and the Kolmogorov-Smirnov test. Comparisons between normally distributed continuous variables were performed using a T test for independent variables. Continuous variables without normally distribution were compared through a non-parametric test (Mann–Whitney U test). The Chi-square test was used for categorical variables. First, comparisons were made between controls and the entire KD group; second, comparisons were made between controls and KD patients with normal GLS. Intra- and interobserver variability, expressed as the mean percentage error (absolute difference/mean), was assessed on ten randomly selected subjects. Interclass coefficients (ICCs) were then calculated (22). Statistical significance was defined as a p-value <0.05. Graphs in figures 2-4 were drawn by using Past software (version 4.02).
